# Racial Disparities in Prostate Cancer Incidence, Biochemical Recurrence, and Mortality

**DOI:** 10.1155/2011/716178

**Published:** 2011-12-13

**Authors:** Cathryn H. Bock, Isaac Powell, Rick A. Kittles, Ann W. Hsing, John Carpten

**Affiliations:** ^1^Department of Oncology, Wayne State University, Detroit, MI 48201-1379, USA; ^2^Department of Urology, Wayne State University, Detroit, MI 48201, USA; ^3^Department of Medicine, University of Illinois at Chicago, Chicago, IL 60607-4067, USA; ^4^Division of Cancer Epidemiology and Genetics, National Cancer Institute, Bethesda, MD 20892-7335, USA; ^5^Integrated Cancer Genomics Division, The Translational Genomics Research Institute (TGen), 445 N. Fifth Street, Phoenix, AZ 85004, USA

Prostate cancer incidence and mortality rates vary widely among populations, with the highest documented rates among American and Caribbean men of African descent and the lowest rates in Asian populations. It is likely that these differences can be attributed to variation in genetics, environmental exposures, access to health care, screening patterns, and treatment patterns; however, the reasons for these differences have not been fully elucidated. 

This special issue includes eight original research articles that provide details regarding racial differences in incidence rates and mortality rates and pathological features and which help us to achieve a better understanding of environmental and clinical reasons for these disparities. Two of the articles examine the validity of prognostic indicators and treatment recommendations in populations external to those in which nomograms and treatment protocols were developed. 

The first research article, “*Prostate cancer incidence rates in Africa,*” characterizes incidence rates within Sub-Saharan Africa populations. Despite differences in data availability and quality across the various locations included, the authors provide strong evidence that incidence rates vary considerably within Africa and that incidence rates are rising in several Sub-Saharan Africa countries. The reported incidence rates in Africa are much lower than those among African American men but are similar to incidence rates of *distant-stage* prostate cancer in African American men.

The next two articles address issues in prostate cancer rates in Caribbean nations. A. J. M. Hennis et al. describe prostate cancer incidence and mortality rates in Barbados and compare these with rates among several other populations in “*Prostate cancer incidence and mortality in Barbados, West Indies*.” In general, rates in Barbados do not differ from rates in other Carribbean populations and are lower than those reported in African Americans. In “*Environment as a potential key determinant of the continued increase of prostate cancer incidence in Martinique*,” D. Belpomme and P. Irigaray identified higher prostate cancer incidence rates in Martinique compared with those in France, Sweden, the USA, and the UK. Reasons for incidence rate differences were examined using an ecological study approach, and evidence favored genetic, pesticide exposure, or gene-environment interaction explanations for rate differences, with no evidence observed for a role of life expectancy or diet on differences in incidence rates. 

Important pathological differences in disease characteristics by race are described in the fourth paper, “*A retrospective study on pathologic features and racial disparities in prostate cancer*.” S. A. Bigler et al. report differences in the prevalence of key histologic and clinical features between African American and Caucasian men at times of biopsy, diagnosis, and prostatectomy. These differences include younger age, higher cancer detection rate, higher Gleason score, more bilateral prostate involvement, larger prostate size, and greater tumor volume among African American men compared with Caucasian men. Differences in tumor evolution described include increased risk of diagnosis of prostate cancer associated with diagnosis of high-grade prostatic intraepithelial neoplasia (HGPIN) or atypical small acinar proliferation (ASAP) among African American men and shorter time from noncancerous biopsy to diagnostic biopsy among men with HGPIN, with ASAP, or of African American race.

In the fifth article, “*The metabolic syndrome and biochemical recurrence following radical prostatectomy*” by J. M. Post et al., hypertension was the most common metabolic feature in both African American and Caucasian prostate cancer cases, with a significantly greater presence among African American men with prostate cancer. Metabolic syndrome was associated with a 50% increase in risk of biochemical recurrence (BCR), and hypertension was associated with a two-fold increase in risk of BCR among both African Americans and Caucasians.

In the sixth article, “*Prostate cancer severity associations with neighborhood deprivation*,” C. M. Ziegler-Johnson et al. report associations between several measures of neighborhood and increased prostate cancer severity in neighborhoods in Southeastern Pennsylvania, with the greatest evidence for association observed within African Americans.

In the seventh article, “*Racial/ethnic patterns in prostate cancer outcomes in an active surveillance cohort,*” J. Cullen et al. compare secondary treatment and overall survival by race/ethnicity in a cohort of men followed on an active surveillance protocol. While black patients were more likely to undergo secondary treatment, no racial differences in overall survival were detected. Given current concerns in the field regarding the overtreatment of prostate cancer and whether active surveillance is appropriate in black men, these findings provide reassuring evidence that, at least when secondary treatment availability is equal, there are no differences in overall survival by race.

In the eighth article, “*Development and external validation of a nomogram predicting the probability of significant Gleason sum upgrading among Japanese patients with localized prostate cancer*,” T. Imamoto et al. describe a nomogram for predicting Gleason sum upgrading from biopsy to radical prostatectomy with better validity in Japanese men than a previous nomogram developed in a Western population. Given that approximately one-fifth of Japanese men diagnosed with prostate cancer at biopsy will experience Gleason sum upgrade, this nomogram may aid clinicians in identifying patients at higher risk of upgrade.

*Cathryn H. Bock* *Cathryn H. Bock* *Isaac Powell* *Isaac Powell* *Rick A. Kittles* *Rick A. Kittles* *Ann W. Hsing* *Ann W. Hsing* *John Carpten* *John Carpten*

